# Salamander *Hox* clusters contain repetitive DNA and expanded non-coding regions: a typical *Hox* structure for non-mammalian tetrapod vertebrates?

**DOI:** 10.1186/1479-7364-7-9

**Published:** 2013-04-05

**Authors:** Stephen Randal Voss, Srikrishna Putta, John A Walker, Jeramiah J Smith, Nobuyasu Maki, Panagiotis A Tsonis

**Affiliations:** 1 Department of Biology, University of Kentucky, Lexington, KY, 40506, USA; 2 Spinal Cord and Brain Injury Research Center, University of Kentucky, Lexington, KY, 40506, USA; 3 Institute of Protein Research, Osaka University, 3-2 Yamadaoka, Suita-Shi, Osaka, 565-0871, Japan; 4 Department of Biology, University of Dayton, Dayton, OH, USA

**Keywords:** *Hox*, Salamander, Genome, Evolution

## Abstract

*Hox* genes encode transcription factors that regulate embryonic and post-embryonic developmental processes. The expression of *Hox* genes is regulated in part by the tight, spatial arrangement of conserved coding and non-coding sequences. The potential for evolutionary changes in *Hox* cluster structure is thought to be low among vertebrates; however, recent studies of a few non-mammalian taxa suggest greater variation than originally thought. Using next generation sequencing of large genomic fragments (>100 kb) from the red spotted newt (*Notophthalamus viridescens*), we found that the arrangement of *Hox* cluster genes was conserved relative to orthologous regions from other vertebrates, but the length of introns and intergenic regions varied. In particular, the distance between *hoxd13* and *hoxd11* is longer in newt than orthologous regions from vertebrate species with expanded *Hox* clusters and is predicted to exceed the length of the entire *HoxD* clusters (*hoxd13*–*hoxd4*) of humans, mice, and frogs. Many repetitive DNA sequences were identified for newt *Hox* clusters, including an enrichment of DNA transposon-like sequences relative to non-coding genomic fragments. Our results suggest that *Hox* cluster expansion and transposon accumulation are common features of non-mammalian tetrapod vertebrates.

## Background

Bilaterian body plans are determined in part by DNA transcription factors called *Hox* genes [1-4]. Excepting fish, vertebrate *Hox* genes are ordered among four unlinked clusters that each span relatively short segments of genomic DNA (generally 100–200 Kb). The arrangement of *Hox* genes on chromosomes is co-linear with their pattern of transcription along the anterior-posterior and proximal-distal body axes during embryonic development [5,6]. The organization and structure of *Hox* gene clusters and associated non-coding regulatory elements are mostly conserved across vertebrates [7,8]. However, as genomic studies extend to non-genetic model organisms, variations in *Hox* cluster structure are being discovered, including variations in gene number, repetitive sequence content, cluster length, and non-coding sequence conservation [9-15]. These variations suggest that the evolution of *Hox* cluster structure may correlate with phylogeny, unique modes of vertebrate development, and/or derived morphological characteristics.

In tetrapod vertebrates, stereotypic patterns of *Hox* expression are observed along the proximal-distal axes of developing limbs [16]. In most species, *Hox* developmental genetic programs are only expressed during limb development. However, salamanders reactivate *Hox* gene expression throughout life to correctly pattern tissues within regenerating limbs [17-21]. While some patterns of *Hox* expression in regenerating limbs recapitulate the expression pattern in developing limbs, spatial and temporal differences are observed [18-21]. This raises the possibility that salamander *Hox* clusters may contain non-coding elements that uniquely regulate post-embryonic, tissue regeneration; such elements may not be expected within *Hox* clusters of vertebrates incapable of limb regeneration. There is another reason to suspect that salamander *Hox* clusters may differ from other vertebrate taxa—salamanders as a group have extremely large genomes. An average sized salamander genome is approximately 10× larger than the *Homo sapiens* genome; some salamanders have genomes that are 30× larger [22]. This larger genome size is reflected in the structure of genes, as salamander introns are longer on average than orthologous introns in other vertebrates [23,24].

Belleville et al. [25] reported that two pairs of adjacent *Hox* cluster genes from the red spotted newt (*Notophthalamus viridescens*) presented highly conserved coding and non-coding sequences relative to orthologous mammalian *Hox* sequences. These results suggested that *Hox* cluster evolution is constrained even within the context of a very large vertebrate genome (>20 pg/haploid nucleus) [22]. However, the results that we present below show that newt *Hox* clusters are more variable than originally thought. Sequencing of large genomic fragments (>100 Kb) reveals regional variation in length across newt *Hox* cluster regions and higher proportions of DNA transposon-like sequences within *Hox* introns and intergenic sequences than non-coding genomic regions. Our results show that expanded non-coding regions and relatively high repetitive DNA sequence content are typical of *Hox* clusters in amphibians and other non-mammalian tetrapod vertebrates.

## Results and discussion

### BAC library screening, sequencing, assembly, and annotation

**Figure 1:**
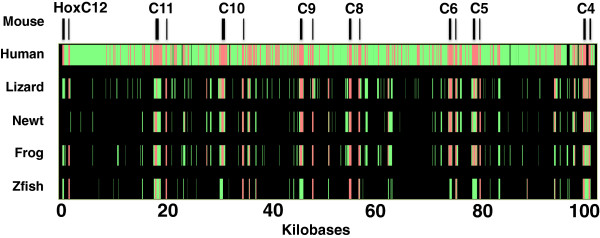
**Multispecies alignment of orthologous *HoxC *genomic regions among selected vertebrate species.** The black bars at the top of the figure show the positions of exons in the mouse sequence, which was used as the reference sequence for alignment. Red bars indicate strongly aligned regions—at least 100 bp in length without a gap and >70% nucleotide identity. Green bars indicate all other aligned regions. Zfish, zebrafish.

**Figure 2:**
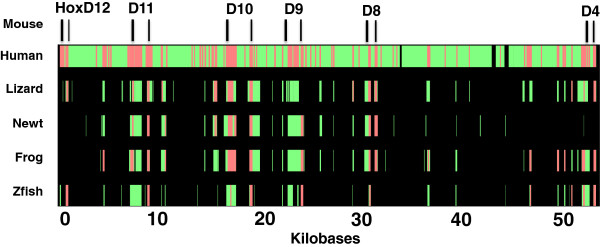
**Multispecies alignment of orthologous *HoxD *genomic regions among selected vertebrate species.** The black bars at the top of the figure show the positions of exons in the mouse sequence, which was used as the reference sequence for alignment. Red bars indicate strongly aligned regions—at least 100 bp in length without a gap and >70% nucleotide identity. Green bars indicate all other aligned regions. Zfish, zebrafish.

A bacterial artificial clone (BAC) library of 41,472 clones was constructed for newt, and pools were screened by polymerase chain reaction (PCR) to identify clones that contained *Hox* genes. Two BACs containing *HoxC* (NV_H3_75P19; [GenBank:JF490017.1]) and *HoxD* (NV_H3_85F1; [GenBank:JF490018.1]) orthologs and two additional BACs containing only non-coding genomic DNA (NV_H3_28J3; [GenBank:JF490019.1] and NV_H3_32L5; [GenBank:JF490020.1]) were purified and sequenced to an average depth of 220 bp sequence reads per nucleotide position. The reads for NV_H3_75P19 were assembled into three large contigs with the breaks occurring between *hoxc5* and *hoxc4*, and a position 3^′^ of *hoxc4*. The reads for NV_H3_28J3 were re-assembled into two large contigs with the break occurring 5^′^ of *hoxd11*. The reads for the BACs that contained non-coding genomic DNA generated more than three contigs and could not be ordered; these were randomly concatenated for analyses described below. BLASTx searches revealed that the BACs containing *Hox* sequences contained some, but not all of *Hox* gene members from each cluster: NV_H3_75P19 contained *hoxc11*, *hoxc10*, *hoxc*9, *hoxc*8, *hoxc*6, *hoxc*5, and *hoxc*4, and NV_H3_85F1 contained *hoxd11*, *hoxd10*, *hoxd*9, and *hoxd*8. The order of newt *Hox* genes was conserved relative to orthologs in other vertebrate genomes, as were coding and non-coding sequences, and exon/intron boundaries (Figures 1 and 2). High sequence identity was observed for *Hox* genes, which is typical of transcription factors that function in highly conserved developmental pathways. Conserved non-coding sequences (CNS) were identified from regions flanking *Hox* exons; these likely correspond to enhancer elements and non-coding RNAs that function in the regulation of *Hox* gene expression. For example, two CNSs that were identified downstream of newt *hoxd11* (40 kb) correspond to enhancer elements VIII and IX from Gerard et al. [26], and a CNS upstream (3 kb) of newt *hoxc8* corresponds to an enhancer from Shashikant et al. [27]. Also, a CNS identified downstream of *hoxc10* (28 kb) corresponds to human miRNA-196a, and a canonical miR-196a seed-pairing site is predicted 268 bp from the end of newt *hoxd8*[28]. Thus, elements that are known to regulate *Hox* gene functions in other vertebrate species show identity to sequences in newt *Hox* genomic regions.

**Figure 3:**
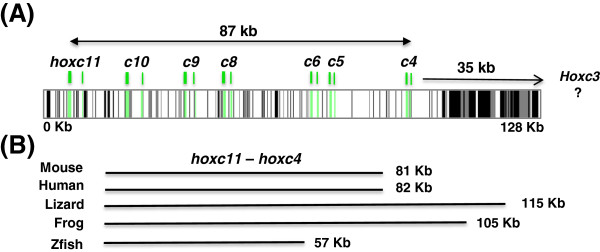
**Structure and repetitive sequence content of newt *HoxC*, and total length of orthologous *hoxc11–c4*.** (**A**) The structure and repetitive sequence content of newt *HoxC*. Green bars indicate the positions of exons, gray bars indicate the positions of RepBase repeats, and black bars indicate the positions of unique newt repetitive sequences. (**B**) The total length of orthologous *hoxc11*–*c4* non-coding segments from representative vertebrates.

**Figure 4:**
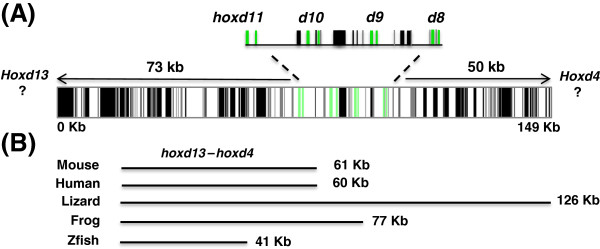
**Structure and repetitive sequence content of newt *HoxD *and total length of orthologous *hoxd11–d8.*** (**A**) The structure and repetitive sequence content of newt *HoxD*. Green bars indicate the positions of exons, gray bars indicate the positions of RepBase repeats, and black bars indicate the positions of unique newt repetitive sequences. (**B**) The total length of orthologous *hoxd11*–*d8* non-coding segments from representative vertebrates.

While the general organization of newt *Hox* genes was conserved relative to other vertebrates, extensive variation was observed in the lengths of intergenic and intronic sequences (Table 1; Figures 3 and Figure 4). The length of the newt *hoxc11*-*c4* region was longer than orthologous mammalian (*H. sapiens*, *Mus musculous*) and zebrafish (*Danio rerio*) regions, but shorter than regions from *Anolis carolinensis* (lizard) and *Xenopus tropicalis* (frog), which are known to have expanded *HoxC* clusters [13]. While newt *HoxC* introns were also longer than mammalian and fish introns, frog and lizard introns also exceed the length of their mammalian counterparts. This supports the idea that salamander genes typically contain long introns [23,24], although we did not observe the same pattern for *HoxD* genes. While long lizard and frog *HoxD* introns were observed, newt *hoxd11*–*9* introns were typically shorter than orthologous mammalian introns. Thus, while non-mammalian tetrapod vertebrates, and especially the anolis lizard, have *Hox* genes with long introns, relative intron length varies among paralogous members of newt *HoxD* and *HoxC* clusters.

**Table 1:** **Species comparison of *HoxC *and *HoxD *intron lengths**

**Gene ID**	**Human**	**Mouse**	**Lizard**	**Frog**	**Newt**	**Zebrafish**
*hoxc11*	1257	1261	2025	1334	*2907*	1368
*hoxc10*	3158	3189	*4591*	2498	3211	1766
*hoxc9*	1703	1704	2139	1560	*2225*	1059
*hoxc8*	1368	1347	*2075*	1619	1393	1265
*hoxc6*	733	728	902	763	*1283*	610
*hoxc5*	701	692	*927*	713	753	818
*hoxc4*	488	475	*1006*	448	502	520
*hoxd11*	770	735	*1667*	748	675	863
*hoxd10*	1375	1366	1628	*1980*	1233	715
*hoxd9*	*348*	346	–	341	345	316
*hoxd8*	373	395	*2474*	1733	434	–

The longest intron is italicized for each *Hox* gene.

In annotating *HoxD* cluster genes, we discovered that *hoxd11* was located approximately 73 kb from the terminus of NV_H385F1. This distance, which provides a minimum estimate to the expected position of *hoxd13* (*hoxd12* is not known for amphibians [15,26]), predicts the newt *hoxd13*–*11* segment to be > 4.5× and 1.5× longer than orthologous *HoxD* regions from frog and lizard. It also exceeds the length of *hoxd11*–*13* segments in the coelacanth and a caecilian amphibian (*Typhlonectes natans*) [29], which until this study was thought to be longest among vertebrates (Figure 4). While it is possible that the expanded region is explained by an evolutionary loss of the newt *hoxd13* gene, this seems unlikely because *hoxd13* orthologs are known for related salamanders [15], and we did not detect the presence of a pseudogene nucleotide signature. Because expanded *Hox* clusters have been shown for a representative caecilian [26] and anuran species [13], parsimony suggests the expansion of the *hoxd11*–*13* region to be a shared derived characteristic of amphibians, with convergent expansion of the same region in lizard.

### Interspersed repeat sequences in BACs

Previous studies have shown that interspersed repetitive DNA sequences are rarely observed within *Hox* clusters of mammals and some reptiles, but are more abundant in species with expanded *Hox* clusters [15,26]. To test this idea, we searched *Hox* and non-*Hox* genomic clones for repeats that are catalogued in RepBase (Genetic Information Research Institute, Mountain View, USA) [30], and also aligned genomic sequences using MultiPipmaker [31] to identify direct and indirect repeats unique to the newt. In many cases, we found that both approaches identified repetitive sequences for the same segments of DNA; however, more newt specific repeats were identified overall (Additional file 1: Table S1). The annotated (i.e., RepBase) interspersed repetitive sequence content of *HoxC* and *HoxD* genomic sequences was approximately two to three times lower than the content of the two, non-protein coding genomic clones (Table 2). Considering annotated and newt-specific repeats, 77% of the non-coding genomic sequence was identified as repetitive, compared to 24% and 32% for *HoxC* and *HoxD* sequences (Additional file 1: Table S1). These results suggest that the fixation probability for repetitive element accumulation is lower for *Hox* clusters, presumably because these regions are evolutionarily constrained by the functional sequences they encode. Repeats were more frequent in regions flanking genes, with the large intergenic regions flanking terminal *Hox* loci showing the greatest accumulation (Figures 3 and 4). Repeats were predicted for introns, and a higher density of DNA transposon-like sequences were predicted within *HoxC* and *HoxD* clusters than within non-coding genomic clones. Interestingly, the enrichment of DNA transposon-like sequences was about 20-fold for *HoxC* but only 2-fold for *HoxD* (Table 2). While this may reflect sampling bias, the more expanded of the two newt *Hox* clusters does not contain a higher proportion of DNA transposon-like sequences; instead, *HoxD* contains a moderately higher proportion of long interspersed retroelement-like sequences, simple repeats, and newt-specific repeats. Observation of a higher frequency of DNA transposon-like sequences, within arguably a more functionally constrained *HoxC* cluster, suggests an insertion bias for *Hox* genic regions. While this speculation awaits further study, our results support the idea that repetitive sequences, and in particular DNA transposon-like sequences, are more abundant within *Hox* clusters of non-mammalian tetrapod vertebrates [13] than is indicated by analysis of mammalian *Hox* clusters.

**Table 2:** Percent coverage of salamander genomic sequences by newt-specific and RepBase repetitive elements

	***HoxC***	***HoxD***	**Non-coding**
Newt-specific repeats	24	32	77
Total RepBase repeats	5.34	4.25	13.34
Long terminal repeat retrotransposons	2.75	2.70	9.50
Non-long terminal repeat retrotransposons	2.30	3.92	2.42
DNA transposons	2.54	0.32	0.12
Unclassified	0.04	0.00	1.30
Satellites	0.00	0.50	0.31
Simple repeats	0.88	1.88	0.87
Low complexity	0.62	0.38	0.31

## Conclusions

Salamander *Hox* genomic regions show elements of conservation and diversity in comparison to other vertebrate species. Whereas the structure and organization of *Hox* coding genes is conserved, newt *Hox* clusters show variation in the lengths of introns and intergenic regions, and the *hoxd13*–*11* region exceeds the lengths of orthologous segments even among vertebrate species with expanded *Hox* clusters. We posit that the *hoxd13*–*11* expansion predated a basal salamander genome size increase that occurred approximately 180 million years ago [32] as it is preserved in all three extant amphibian groups. Over more recent timescales, additional evidence supports the idea that *Hox* clusters are amenable to structural evolution: there is variation in the lengths of introns and intergenic regions, relatively high numbers of repetitive sequences, and non-random accumulations of DNA transposons in newts and lizards. The non-random accumulation of DNA-like transposons could potentially alter developmental programming by creating sequence motifs for transcriptional regulation [33-35]. Overall, available data from several non-mammalian tetrapods suggest that *Hox* structural flexibility is the rule, not the exception. We speculate that such flexibility may contribute to developmental variation across non-mammalian taxa, both in embryogenesis and during the re-deployment of *Hox* genes during post-embryonic developmental processes, such as metamorphosis and regeneration.

## Methods

### BAC library construction, screening, and sequencing

The Clemson University Genomics Institute constructed a BAC library from partially restriction digested and size-selected genomic DNA that was isolated from the erythrocytes of a single *Notophthalamus viridescens* female (University of Dayton Institutional Animal Care and Use Committee Protocol # 011–12). A total of 41,472 clones were arrayed in 108 × 384 well plates. Superpools of clones were made by combining clones from twelve 384 well plates into a single pool. DNA was extracted from 400 ml of overnight cultures of superpools using the Plasmid MaxiPrep kit (Qiagen, Valencia, CA, USA), and the DNA pellet was re-suspended in 250 μl of water. PCR primers for newt *hoxc10* (forward: CAAAGAGAAAACGCGGAAAG; reverse: CGATACCGTCCCTTCCATAA) and *hoxd10* (forward: TTTCCATTGTCGGTTTTTCC; reverse: TCCTACCACGGACATTACCC) were used to identify two *Hox* gene-containing BACs and two BACs that did not contain protein-coding sequence. The four clones were grown in 400 ml L-broth, and DNA was isolated using the Qiagen Large Construction Kit (Qiagen); genomic DNA contamination was reduced using Plasmid–Safe DNAse treatment (Epicentre Biotechnologies, Madison, USA). The Roche GS FLX Titanium platform (Basel, Switzerland) was used to sequence BACs; the work was accomplished by the staff of the University of Iowa Sequencing Core Facility. The termini of BAC inserts were end-sequenced using Sanger technology and ABI Big-Dye 3.1 (Invitrogen, Grand Island, USA).

### DNA sequence assembly and annotation

Sequences were screened to trim vector, adapters, and contaminating *Escherichia coli* sequences. After an initial assembly using GS De Novo Assembler (454 Life Sciences, Branford, USA), contigs and singletons were assembled further using DNASTAR SeqMan (DNASTAR, Inc., Madison, USA). Contiguous sequences of assembled BACs were searched (*blastn*) against salamander expressed sequence tagged contigs at Sal-Site [36]; non-redundant nucleotide and protein databases at NCBI (*blastx* and *tblastp*) [37] were used to identify and annotate gene regions. For multispecies comparisons, genomic sequences for *H. sapiens* (GRCh37.10), and *M. musculus* (GRCh38.1), were obtained from NCBI. *Anolis carolinesis* (AnoCar 2.0) *and D. rerio* (Zv9) were obtained from Ensembl [38]. *X*. *tropicalis* (build 7.1) was obtained from Xenbase [39]. Sequences were aligned using MultiPipMaker [28]. Annotated repeats were identified by searching re-assembled BAC clones against all deposited repeats in RepBase [30]. Newt-specific repeats were identified using MultiPipmaker [28] by aligning re-assembled BAC clones against each other and by performing self-self BAC alignments. The “search both strands” and “high sensitivity” options were used in MultiPipmaker to identify significantly similar non-coding sequences that are located to different positions either within or between BACs. The terminal base pair positions for these alignments were recorded to denote the positions of repetitive sequences within BACs. If the two repeats occurred within 50 bp of each other, they were compiled as a single repetitive sequence with the most terminal base positions denoting the repeat span. The base pair coordinates for newt-specific repetitive sequences were combined with base pair coordinates for RepBase repetitive sequences to generate an underlay file (Additional file 1: Table S1), and this was used to create maps of repetitive elements for the *HoxD* and *HoxC* genomic regions.

## Competing interest

The authors declare that they have no competing interests.

## Authors’ contributions

SRV, JJS, NM, and PAT conceived and designed the experiment, and analyzed and interpreted the results. SP assembled sequence reads corresponding to BACs and analyzed and annotated the sequence data. JAW performed PCR, isolated BACs for sequencing, and analyzed and annotated the sequence data. SRV drafted the manuscript. All authors read and approved the final manuscript.

## Supplementary Material

Additional file 1: Table S1 Base pair coordinates for RepBase and newt-specific repeats identified from BAC clones with *HoxC* and *HoxD* genomic regions. Click here for file
